# Additional circular intercostal space created by bifurcation of the left 3rd rib and its costal cartilage: a case report

**DOI:** 10.1186/1752-1947-7-6

**Published:** 2013-01-08

**Authors:** Naveen Kumar, Anitha Guru, Jyothsna Patil, Swamy Ravindra, Satheesha Nayak Badagabettu

**Affiliations:** 1 Department of Anatomy, Melaka Manipal Medical College (Manipal Campus), Manipal University, Manipal, Karnataka State, 576104, India

**Keywords:** Bifid costal cartilages, Bifid rib, Circular intercostal space, Rib bifurcation

## Abstract

**Introduction:**

In the thorax there are normally 11 pairs of intercostal spaces: the spaces between adjacent ribs. The intercostal spaces contain intercostal muscles, intercostal nerves and vessels.

**Case presentation:**

During a routine dissection for undergraduate medical students, we observed a variation involving the left 3rd rib and 3rd costal cartilage in the cadaver of a man of Indian ethnicity aged about 65 years. The left 3rd rib and its costal cartilage were bifurcated at their costochondral junction enclosing a small circular additional intercostal space. Muscle tissue covered by deep fascia was present in this circular intercostal space. The muscle in the circular intercostal space received its nerve supply from a branch of the 2nd intercostal nerve.

**Conclusions:**

Knowledge of such variations is helpful to surgeons operating on the anterior thoracic wall involving ribs and intercostal spaces. Knowing the possibility of the presence of an additional space between normal intercostal spaces can guide a surgeon through to a successful surgery.

## Introduction

The ribs are essential structures of the osseous thorax and provide information that aids in the interpretation of radiologic images. A rib develops from the costal process of the developing thoracic vertebra through endochondral ossification [1]. A bifid rib or bifurcated rib is an unusual malformation and is rarely observed in the clinical scenario. It is a congenital abnormality of the rib cage and associated muscles and nerves in which the sternal end of the rib is cleaved into two. It is usually unilateral and asymptomatic but it can present as an isolated abnormality or be associated with pathologic malformations such as jaw cysts and basal cell nevus syndrome [2]. The reported cases of bifid rib were found in X-ray investigations or some symptomatic patients, with few previous reports involving cadavers.

## Case presentation

**Figure 1:**
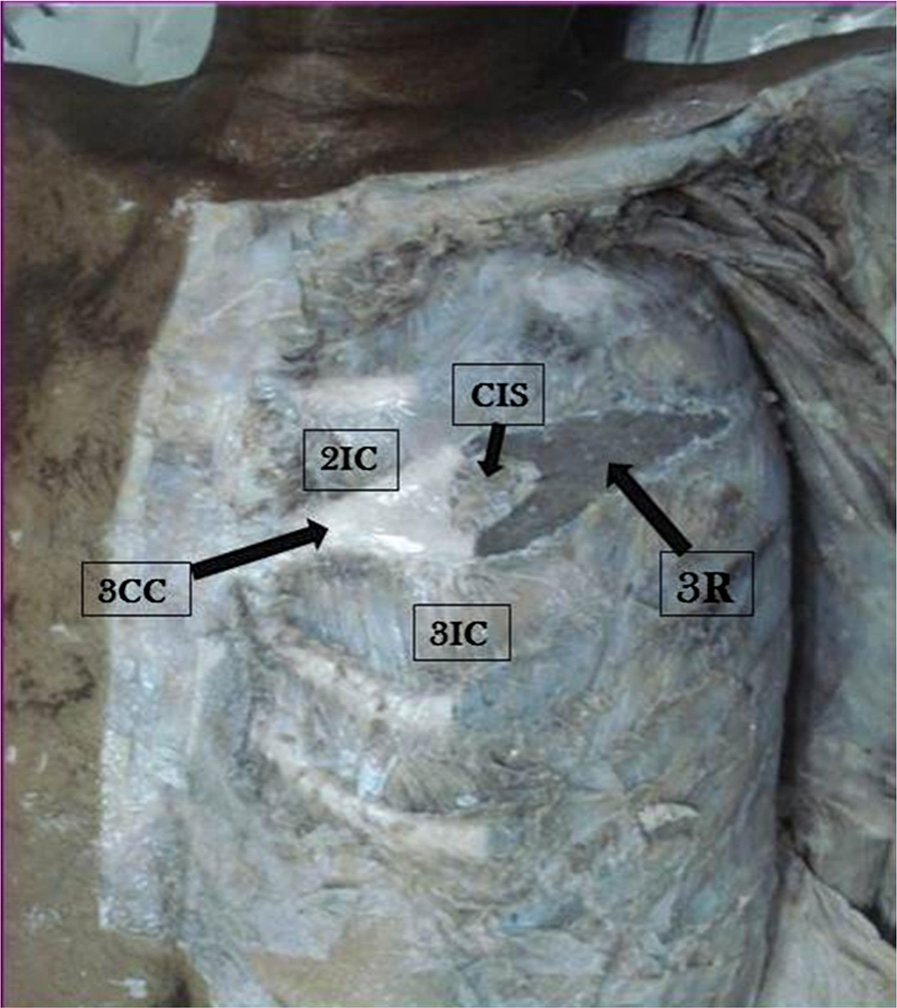
**Dissection of left side of the thorax showing circular intercostal space intervening between second and third intercostal spaces, encircled by bifid third rib and bifid third costal cartilage.** CIS, circular intercostal space; 2IC, second intercostal space; 3CC third costal cartilage; 3IC, third intercostal space; 3R, third rib.

During the cadaveric dissection of the thorax of a man of Indian ethnicity aged about 65 years, we observed a variation of bifid appearance involving the left 3rd rib and 3rd costal cartilage enclosing an additional circular intercostal space. In the current case, the left 3rd costal cartilage bifurcated 3.4cm from the lateral border of the sternum. The left 3rd rib bifurcated at its sternal end and articulated with the corresponding limbs of the bifid costal cartilage. Hence there were two costochondral junctions instead of one (Figure 1). The space between the upper and lower divisions was circular, and was situated between the 2nd and 3rd intercostal spaces. This additional intercostal space contained intercostal muscles that were covered by fascia.

Although the additional intercostal space was too small to allow the muscular layers to be distinguished, a careful dissection was made to separate the layers of muscles and their innervations. They were supplied through the collateral branch of the 2nd intercostal nerve. The collateral branch of the 2nd intercostal nerve took a deep course to give its twigs to the muscles in the additional intercostal space. The size of the 2nd intercostal space was reduced due to bifurcation of the rib; however, the 3rd space was of normal size. Remaining parts of the 3rd rib and 3rd costal cartilage were observed to be normal. This phenomenon was unilateral and observed in the cadaver of a man.

## Discussion

The ribs are essential structures of the osseous thorax and provide information that aids in the interpretation of radiologic images. The transverse appearance of the thorax and costal shape are landmarks in the detection of thoracic deformities like pectus excavatum and barrel-shaped thorax.

Anatomic rib variants include developmental deformities, cervical rib, and short rib which may mimic true rib diseases. Apart from this there may be rare occasions where unusual variations are encountered; for example, the costal cartilage and adjacent portions of the body of the rib may be occasionally replaced by fibrous tissue, two adjacent ribs may be completely fused, or the bodies of two or more ribs may be joined by fusion [3]. Many authors opine that congenital abnormalities of the rib are relatively common, particularly bifid ribs.

**Figure 2:**
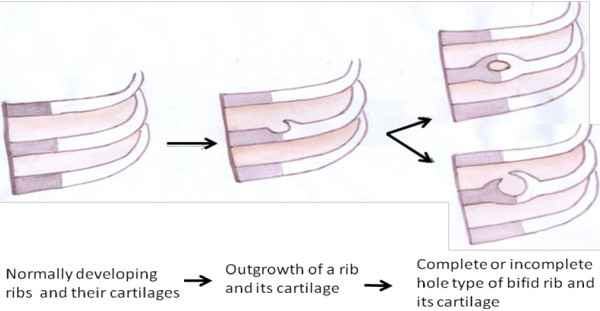
Developmental course of bifid rib and its associated structures.

The anatomy of the bifid rib has a great effect on its development. Osawa *et al*. [4,5] in their two separate studies observed a bifid phenomenon in the 3rd, 4th and 5th ribs. The authors also reported a rare case of bilateral bifid ribs. The possible course of bifid rib and its cartilage resulting in an additional intercostal space can be illustrated by a schematic diagram (Figure 2).

An extensive study of chest photo roentgenograms by Etter *et al*. [6] showed that bifid ribs are more common in males than females, and occur most frequently in the third and fourth ribs. According to them the degree of incidence of bifid ribs are in the order of third>fourth>fifth>sixth>second. Bloomberg [7] suggests that one must be careful to differentiate this condition from the fusion of two ribs, which may give the appearance of a bifurcate rib.

Various incidences and types of numerical and structural abnormalities of the ribs have been reported. According to Lim *et al*. [8], incidences of bifurcated ribs are slightly more common on the right side than on the left. They conducted a study based on X-rays and reported the incidence of rib anomalies in Koreans to be 2.8%. They also stated that bifid ribs were the most common type of rib anomaly in Koreans and they account for 1.7% of rib anomalies in the general population and 59.6% of cases with total costal anomalies.

Bifid ribs associated with pathologic malformations such as Gorlin–Goltz syndrome [9,10] and malignancy in childhood usually occur in the young [11], and might be characteristic of multiplicity of the bifid rib on the same side.

In older people, the bifid rib when it is not associated with other disease may present few clinical problems. However, knowledge of bifid ribs is necessary for the differential diagnosis with other diseases, such as tumors of the chest wall or costal fracture, because the various types of bifid rib present with diverse appearances on normal chest X-rays [12].

The ribs and the intercostal spaces provide important surface marking for various physical examination procedures and clinical procedures. Hence it is imperative that the radiologist be familiar with normal rib anatomy, normal rib variants, and the radiologic appearance of the ribs to prevent misdiagnosis [13].

An additional intercostal space and its associated contents are often symptomless but occasionally the effects of this neuroskeletal anomaly, which includes respiratory difficulties, may show.

## Conclusion

The present case is different from previously reported bifid ribs found in cadavers because this case was observed in the left side of the thoracic cage immediately lateral to the sternum. Much attention is paid in such cases because the pulmonary trunk lies deep to this circular intercostal space. Hence the knowledge of such variation is of utmost importance to surgeons performing thoracic surgeries because such an anomaly could mislead them during their procedures. Careful enumeration of the ribs, however, should preclude this error.

## Consent

Consent was given by the patient for the use of his body for medical education and advancement.

## Competing interests

The authors declare that they have no competing interests.

## Authors’ contributions

NK prepared the manuscript. AG compiled the data collection. JP dissected the cadaver. SR contributed in the preparation of the specimen. SNB edited the manuscript. All authors read and approved the final manuscript.
